# Effectiveness of a Learning Path in the Acquisition of Evidence-Based Practice Competencies by Nurses: A Protocol for a Systematic Review

**DOI:** 10.3390/nursrep15120439

**Published:** 2025-12-10

**Authors:** Catarina Pinto, Cristina Barroso Pinto, Maria Marques, Liliana Mota

**Affiliations:** 1 Critical Care Unit, Local Health Unit of Aveiro’s Region, 3814-501 Aveiro, Portugal; 2 Comprehensive Health Research Centre (CHRC), University of Évora, 7000-801 Évora, Portugal; mcmarques@uevora.pt; 3 Nursing School of Porto, RISE-Health, 4200-072 Porto, Portugal; cmpinto@esenf.pt; 4 Department of Nursing, University of Évora, 7000-811 Évora, Portugal; 5 Department of Nursing, Northern Health School of the Portuguese Red Cross, RISE-Health, 3720-126 Oliveira de Azeméis, Portugal; liliana.mota@essnortecvp.pt

**Keywords:** evidence-based practice, learning paths, nurses, systematic review

## Abstract

**Background/Objectives**: Evidence-Based Practice (EBP) positively impacts health safety and quality while also empowering nursing as a discipline. A useful strategy for promoting EBP is to build learning paths adapted to the individuality of nurses. These elements establish the framework for effective learning, determining the availability of specific content at certain times and influencing the design of learning objects to ensure optimal efficacy in the teaching-learning process. It is essential to identify effective strategies in evidence-based nursing education to advance EBP and thereby enhance the quality and safety of nursing care. This review aims to summarize the evidence on the effectiveness of learning paths in the acquisition of EBP competencies by nurses. **Methods**: A systematic review of the literature will be carried out in accordance with the Joanna Briggs Institute (JBI) methodology for systematic reviews of effectiveness. The results of the review will be reported according to the Preferred Reporting Items for Systematic Reviews and Meta-Analyses protocols (PRISMA-P). The protocol is registered in the PROSPERO database (CRD4202453155). The search will be performed using the EBSCOhost search engine in the following databases: CINAHL Plus, MedicLatina, MEDLINE, Psychology and Behavioral Sciences Collection, Academic Search Complete, eBook Collection, and Education Resources Information Center. The inclusion of studies, data extraction, and analysis will be carried out by two reviewers independently. Disagreements will be resolved by a third reviewer. All studies involving nurses, learning paths, EBP competencies, regardless of geographical area and context, with no time limit or language constraints, will be included. **Results**: Not applicable; this is a protocol. Findings will be synthesized as specified in the Methods. **Conclusions**: This review will provide a better understanding of the effectiveness of a learning path in the acquisition of EBP competencies by nurses. It will also assist in the identification of knowledge gaps in the literature and potential areas for future research and development.

## 1. Introduction

Health institutions and their professionals are constantly faced with challenges whose management requires a high level of quality and efficiency, which is only possible through the use of Evidence-Based Practice (EBP). This refers to the integration of the best scientific evidence with the clinical experience of the professional, and the values and choices of the person at the center of care, according to the available resources [1,2].

The positive impact of EBP is reflected in the promotion of safety, efficiency, and quality of care, the promotion of organizational reliability, the prevention of complications, the promotion of better outcomes for the client, the reduction in costs, and the optimization of health systems [2,3,4,5,6,7,8]. The use of evidence and its translation into nurses’ clinical practice fosters their critical thinking, promotes safety in decision-making, and thus increases the credibility of nursing as a discipline of knowledge [9,10].

Despite the positive impact that EBP has on nurses’ professional development, its effective implementation in nurses’ practice contexts remains a challenge worldwide [8,11,12,13,14]. Current literature alludes to numerous obstacles to the EBP process, including a lack of time, training, knowledge, and skills to carry out research, a lack of mentors, difficulty in critically analyzing scientific articles, difficulty in operationalizing the different stages of the EBP process, a lack of resources, limited support and encouragement from managers, and a high workload [3,8,9,10,13,14,15,16]. With regard to the factors that promote EBP, these are related to the availability of time, resources, and administrative support, investment in continuous and postgraduate training, an organizational culture based on EBP, the existence of EBP mentors, and cooperation between researchers and practice nurses [13,15,16,17].

In order to promote the implementation of EBP, various programs have been designed to foster the development of competencies in EBP by these health professionals [6,17,18,19]. These programs are characterized by training sessions of various formats, frequencies, and durations, aimed at a group of nurses with different expectations, academic degrees, professional experience, and learning styles. As a result, the programs may not fully align with the specific needs and/or individualities of each professional. These programs are part of traditional classroom teaching strategies, which have been widely compared with digital learning strategies in the context of nurse training [20,21]. Digital learning strategies have consistently demonstrated more favorable outcomes [21,22], including the promotion of knowledge acquisition and skills development [20], as well as leadership and problem-solving skills [21].

Technological advances and pedagogical innovation that are characteristic of modern education have resulted in online education and the use of digital technologies becoming an integral part of contemporary education [23,24,25]. In the field of health, its use has grown exponentially in the training of nurses, both in clinical and educational contexts [26,27,28]. In their book entitled “Knowledge Translation in Nursing and Healthcare: A Roadmap to Evidence-informed Practice”, Harrison and Graham [11] state that the sustained implementation of EBP requires the construction of a learning path that integrates nurses’ workflow, which can be achieved through digital means.

Integrating digital teaching tools positively impacts knowledge acquisition and the development of professional skills and attitudes. It improves performance and promotes collaboration, communication, self-confidence, self-efficacy, problem-solving, and decision-making skills. It also optimizes the preparation of students and health professionals (doctors and nurses) for clinical practice [20,21,25,26,28]. Therefore, resorting to a digital learning path enhances learning outcomes in different areas of knowledge and mitigates barriers related to resources, geographical limitations, and time [28].

According to Vanitha et al. [29], building a learning path is a useful strategy that enhances the teaching-learning process and optimizes participants’ performance. According to the authors, a learning path is defined as “the art of organizing content, exercises, and learning activities to achieve learning objectives” [29]. They determine how learning takes place, guiding the learner along a specific path by directing their access to content at certain times and configuring the learning objects in a specific way, thus ensuring the relationship between learning objectives and content [29]. According to Nabizadeh et al. [30] (2020), a learning path is a structured curriculum implementation strategy consisting of an integrated set of educational activities that guide students toward achieving established learning goals. This pedagogical approach provides content, methods, and resources in an orderly and intentional manner to promote gradual and continuous knowledge acquisition. In a digital learning path, students follow a set of activities designed to align with defined educational objectives within a digital tool, application, or platform [31].

Individuality is a crucial aspect to consider when designing learning paths because it can affect the educational process. Each learner is unique, with specific learning needs and abilities, different motivations, personalities, and emotions [29,32]. Moreover, with regard to learning styles, individuals have different preferences in terms of how they perceive, retain, process, and understand information [33]. In 2022, in Brazil, Figueiredo et al. [34] carried out a qualitative study on the learning styles of nurses, reiterating the importance of identifying and characterizing learning styles to enhance knowledge acquisition, skill development, and ultimately, improve healthcare.

Adapting to individual characteristics and personalizing learning paths is crucial for optimizing educational outcomes, stimulating interest, and fostering motivation to learn [29,35]. However, individualization and personalization are among the main challenges today, given that students have different previous experiences, educational goals, and cognitive, social, and contextual constraints. Thus, adapting digital learning paths to each person’s specific needs is essential to ensuring more inclusive and effective educational processes consistent with contemporary educational requirements [30], as well as promoting the development of EBP skills among nurses to provide quality, safe nursing care.

The American Nurses Association defines competency as “an expected and measurable level of nursing performance that integrates knowledge, skills, abilities, and judgment based on established scientific knowledge and expectations for nursing practice” [36] (p. 86). Competency in nursing is a complex and dynamic concept that is constantly evolving [37]. It brings together the essential attributes that enable nurses to perform their roles effectively and ensure that they provide safe, high-quality care [38]. EBP competencies are embodied in a set of 24 competencies: 13 for registered nurses and 11 for advanced practice nurses, as identified by Melnyk et al. [38] in a 2014 study.

The research carried out revealed that there is no systematic review addressing the effectiveness of a learning path in the acquisition of EBP competencies by nurses, thus highlighting the importance of this study. It seeks to generate new knowledge and contribute to future developments.

The aim of the study was to summarize the evidence on the effectiveness of learning paths in the acquisition of EBP competencies by nurses. This protocol clarifies and defines the conditions for conducting the review to ensure the rigor, clarity, and quality of the process. Two independent reviewers will be responsible for searching, identifying, and selecting studies.

## 2. Materials and Methods

This protocol was developed in accordance with the Preferred Reporting Items for Systematic Reviews and Meta-Analyses Protocols (PRISMA-P) [39]. On 13 April 2024, it was registered in the International Prospective Register of Systematic Reviews (PROSPERO), under registration number CRD42024531556 (https://www.crd.york.ac.uk/PROSPERO/view/CRD42024531556, accessed on 9 December 2025).

This review will include primary empirical studies, qualitative or quantitative (observational or experimental, cross-sectional or longitudinal).

This protocol was developed in March 2024. It is anticipated that the review will be finalized and the results disseminated in December 2025.

This study does not require ethics approval, as the systematic review methodology involves reviewing and collecting data from publicly available materials. The results of this study will advance knowledge of learning paths and their effectiveness in the acquisition of EBP competencies by nurses. This will promote the sustained implementation of evidence in nurses’ clinical practice, resulting in health gains.

### 2.1. Review Questions

This review sets out to answer the following PICO research question: “How effective are learning paths (I) compared to traditional teaching practices (C) in the acquisition of Evidence-Based Practice competencies (O) by nurses (P)?” The same review could also answer the following research question: What elements should be part of the design learning paths?

### 2.2. Identifying Relevant Studies

#### 2.2.1. Population

The inclusion criteria are nurses, regardless of their academic degree. Undergraduate nursing students and nursing technicians are excluded.

#### 2.2.2. Intervention

The review will include studies on learning paths, regardless of the geographical area and context.

#### 2.2.3. Comparison

This review will include studies that compare the results of learning paths with traditional teaching practices.

#### 2.2.4. Primary Result

The main objective will be to summarize the evidence on the effectiveness of learning paths in the acquisition of EBP competencies by nurses.

#### 2.2.5. Study Design

Systematic literature review protocol according to the PRISMA-P [39].

#### 2.2.6. Context

This review will include all studies related to learning paths for nurses, regardless of the context.

### 2.3. Selecting Studies for Analysis

#### 2.3.1. Data Source

The search will be conducted in CINAHL Complete, MedicLatina, MEDLINE Complete, Nursing and Allied Health Collection: Comprehensive, Cochrane Central Register of Controlled Trials, Cochrane Databases of Systematic Reviews, Cochrane Methodology Register, Library, Information Science and Technology Abstracts, Cochrane Clinical Answers, using the EBSCOhost search engine; Scopus, Web of Science, and ProQuest.

#### 2.3.2. Search Terms

The search will include the combination of key concepts according to the research question. Medical Subject Headings (MeSH) terms were used, along with natural language terms, taking into account the existence of important concepts that are not considered MeSH terms. The pilot search strategy was developed using the PubMed/MEDLINE database (Table 1). Then, the strategy will be adapted for each database. There will be no restrictions based on publication year or language; all available studies up to the final search date will be considered.

### 2.4. Data Collection and Analysis

#### 2.4.1. Study Selection

The studies resulting from the search will be exported to the Rayyan application (http://rayyan.qcri.org, accessed on 9 December 2025), to facilitate study screening and selection, and duplicate studies will be removed.

To reduce the risk of bias, two reviewers (CP and LM) will independently read the title, abstract, and keywords and exclude any studies that do not meet the previously defined inclusion criteria. If there is any disagreement, a third reviewer will be consulted.

All the selected articles will be organized using the digital tool ZOTERO^®^ (version 6.0.36), which will facilitate monitoring the review’s progress and maintaining organized data.

The results of the screening at each stage will be presented in the form of a PRISMA flowchart [40].

#### 2.4.2. Data Extraction

Data extraction will be performed using a previously prepared extraction tool, which will include, in addition to information related to the research questions, the authors of the studies, year of publication, country, objective of the study, methods, results, and main conclusions. As mentioned above, this stage will be carried out independently by the same two reviewers (CP and LM), and any disagreement will be clarified by a third reviewer.

#### 2.4.3. Quality Assessment

The quality assessment tools will be the JBI (Joanna Briggs Institute) assessment tools for qualitative and quantitative studies [41,42]. Similarly, two reviewers (CP and LM) will independently carry out this stage, and any disagreement during the quality assessment of the studies will be resolved by a third reviewer. The results of the quality assessment of the studies will be presented.

#### 2.4.4. Data Synthesis Strategy

The data synthesis will be narrative in nature in order to answer the research questions initially defined. For quantitative or mixed-method studies, the narrative synthesis will be conducted following the framework proposed by Popay et al. [43]. For qualitative studies, JBI meta-aggregation [44] will be employed. This approach is internationally recognized as a rigorous method for synthesizing qualitative evidence. Using this combination of methods ensures that each type of study is addressed with the most appropriate synthesis strategy, thereby enhancing the review protocol’s overall robustness and credibility.

A table summarizing the results of each study will be created to facilitate the analysis and discussion process. Information related to the research questions, the authors of the studies, year of publication, country, study objective, methods, results, and main conclusions will be identified.

To optimize the presentation of the data, all authors will participate in this stage.

#### 2.4.5. Assessment of the Quality of the Evidence Produced by the Review

The quality of the evidence produced by the review will be assessed using the GRADE (Grade of Recommendations Assessment, Development and Evaluation) protocol and GRADE-CERQual (Confidence in the Evidence from Reviews of Qualitative research) [45,46,47].

Data integration will be conducted using a parallel approach, as recommended by Hong et al. [48] and by the methodological guidance of the JBI [49]. This approach ensures rigorous analysis of qualitative and quantitative evidence, as well as subsequent comparison of the results.

## 3. Results

The systematic literature review is expected to be completed by the end of December 2025. The results will be presented in narrative form and using summary tables. A PRISMA flow diagram will illustrate the study selection process. The aim is to disseminate the research results by publishing a scientific article in a peer-reviewed, open-access scientific health journal and to participate in prestigious scientific conferences in the field.

## 4. Conclusions

In the current context of growing demand for efficient healthcare, implementing EBP is essential to ensure excellence in care and achieve health gains. However, the gap between research and its practical application persists.

This review will synthesize evidence on the effectiveness of a learning path in the development of EBP competencies, contributing to their development.

Equipped with a professional mindset centered on conceptual logic, nurses use the best evidence to identify human responses that are potentially sensitive to nursing care. Not all professionals possess this ability, which depends on their level of expertise in EBP competencies. Therefore, a learning path tailored to nurses’ individual characteristics will promote the development of their EBP competencies and the sustained implementation of evidence in clinical practice contexts, resulting in undeniable health gains for clients, professionals, and their respective organizations.

The implications for the practice of this work are clear: promoting EBP among nurses through a learning path is a fundamental and innovative strategy for empowering this future healthcare workforce, promoting safety and quality of care, optimizing healthcare system performance, and fostering organizational reliability.

## Figures and Tables

**Table 1:** Search strategy used in PubMed/MEDLINE.

Search	Query	Results Retrieved
**#1**	(((“nurs*”[All Fields] OR “nurs* clinician*”[All Fields] OR “nurs* specialist*”[All Fields] OR (“Nursing”[MeSH Terms] OR “Nursing”[All Fields] OR “nursings”[All Fields] OR “Nursing”[MeSH Subheading] OR “nursing s”[All Fields]) OR “nurs* practitioner*”[All Fields] OR “Nursing staff”[MeSH Terms] OR (“nurs*”[Title/Abstract] OR “nurs* clinician*”[Title/Abstract] OR “nurs* specialist*”[Title/Abstract] OR “Nursing”[Title/Abstract] OR “nurs* practitioner*”[Title/Abstract] OR “Nursing staff”[Title/Abstract])) NOT “licensed practical nurs*”[All Fields]) NOT (“licensed practical nurs*”[Title/Abstract] OR “nursing student*”[Title/Abstract]))	1,113,629
**#2**	(“evidence based practic*”[All Fields] OR “Evidence-based nursing”[All Fields] OR “evidence based nursing practic*”[All Fields] OR “evidence based practic*”[Title/Abstract] OR “Evidence-based nursing”[Title/Abstract] OR “evidence based nursing practic*”[Title/Abstract])	35,859
**#3**	(“aptitude”[MeSH Terms] OR “aptitude”[All Fields] OR “abilities”[All Fields] OR “ability”[All Fields] OR “competenc*”[All Fields] OR “abilities”[Title/Abstract] OR “competenc*”[Title/Abstract] OR “skills”[Title/Abstract]) AND (medline[Filter])	1,435,561
**#4**	(“learning path”[All Fields] OR ((“learning”[MeSH Terms] OR “learning”[All Fields] OR “learn”[All Fields] OR “learned”[All Fields] OR “learning s”[All Fields] OR “learnings”[All Fields] OR “learns”[All Fields]) AND “patways”[All Fields]) OR (“roadmap”[All Fields] OR “roadmapping”[All Fields] OR “roadmaps”[All Fields]) OR “learning strateg*”[All Fields] OR “teaching method*”[All Fields] OR “learning method*”[All Fields] OR ((“educability”[All Fields] OR “educable”[All Fields] OR “educates”[All Fields] OR “education”[MeSH Subheading] OR “education”[All Fields] OR “educational status”[MeSH Terms] OR (“educational”[All Fields] AND “status”[All Fields]) OR “educational status”[All Fields] OR “education”[MeSH Terms] OR “education s”[All Fields] OR “educational”[All Fields] OR “educative”[All Fields] OR “educator”[All Fields] OR “educator s”[All Fields] OR “educators”[All Fields] OR “teaching”[MeSH Terms] OR “teaching”[All Fields] OR “educate”[All Fields] OR “educated”[All Fields] OR “educating”[All Fields] OR “educations”[All Fields]) AND “intervencion*”[All Fields]) OR “educational program*”[All Fields] OR (“educacional”[All Fields] AND “strateg*”[All Fields]) OR “workshop*”[All Fields] OR “training*”[All Fields] OR “program*”[All Fields] OR “course*”[All Fields] OR (“learning path”[Title/Abstract] OR “roadmap”[Title/Abstract] OR “learning strateg*”[Title/Abstract] OR “teaching method*”[Title/Abstract] OR “learning method*”[Title/Abstract] OR “educational program*”[Title/Abstract] OR “workshop*”[Title/Abstract] OR “training*”[Title/Abstract] OR “program*”[Title/Abstract] OR “course*”[Title/Abstract] OR “bootcamp*”[Title/Abstract]))	3,088,706
**#5**	((#1) AND (#2) AND (#3) AND (#4))	1349

## Data Availability

Data is contained within the article.

## Public Involvement Statement

No public involvement in any aspect of this research.

## Guidelines and Standards Statement

This manuscript was drafted against the Preferred Reporting Items for Systematic Review and Meta-Analysis Protocols (PRISMA-P) 2015 statement for systematic review protocols.

## Abbreviations

The following abbreviations are used in this manuscript:

Abbreviation	Full term
CINAHL	Cumulative Index to Nursing and Allied Health Literature
EBP	Evidence-Based Practice
GRADE	Grade of Recommendations Assessment, Development, and Evaluation
GRADE-CERQual	Confidence in the Evidence from Reviews of Qualitative Research
JBI	Joanna Briggs Institute
MEDLINE	Medical Literature Analysis and Retrieval System Online
MeSH	Medical Subject Headings
PRISMA-P	Preferred Reporting Items for Systematic Review and Meta-Analysis Protocols
PROSPERO	International Prospective Register of Systematic Reviews
